# Electrically Parallel Three-Element 980 nm VCSEL Arrays with Ternary and Binary Bottom DBR Mirror Layers

**DOI:** 10.3390/ma14020397

**Published:** 2021-01-14

**Authors:** Nasibeh Haghighi, James A. Lott

**Affiliations:** Faculty II Mathematics and Natural Sciences, Institute of Solid-State Physics, Center of Nanophotonics, Technische Universität Berlin, 10623 Berlin, Germany; nasibeh.haghighi@tu-berlin.de

**Keywords:** VCSEL, vertical cavity surface emitting laser, optical wireless communication, optical interconnects, free-space optical communication, fifth generation (5G), sixth generation (6G)

## Abstract

To meet the performance goals of fifth generation (5G) and future sixth generation (6G) optical wireless communication (OWC) and sensing systems, we seek to develop low-cost, reliable, compact lasers capable of sourcing 5–20 Gb/s (ideally up to 100 Gb/s by the 2030s) infrared beams across free-space line-of-sight distances of meters to kilometers. Toward this end, we develop small arrays of electrically parallel vertical cavity surface emitting lasers (VCSELs) for possible future use in short-distance (tens of meters) free-space optical communication and sensing applications in, for example, homes, data centers, manufacturing spaces, and backhaul (pole-to-pole or pole-to-building) optical links. As a starting point, we design, grow by metal–organic vapor phase epitaxy, fabricate, test, and analyze 980 nm top-emitting triple VCSEL arrays. Via on-wafer high-frequency probe testing, our arrays exhibit record bandwidths of 20–25 GHz, optical output powers of 20–50 mW, and error-free data transmission at up to 40 Gb/s—all extremely well suited for the intended 5G short-reach OWC and sensing applications. We employ novel p-metal and top mesa inter-VCSEL connectors to form electrically parallel but optically uncoupled (to reduce speckle) arrays with performance exceeding that of single VCSELs with equal total emitting areas.

## 1. Introduction

We design, fabricate, and test three-element (triple) electrically parallel 980 nm vertical cavity surface emitting laser (VCSEL) arrays. We employ a new VCSEL epitaxial design that includes a mix of binary (AlAs and GaAs) and ternary (AlGaAs) bottom distributed Bragg reflector (DBR) layers. Binary DBR layers reduce thermal resistance and increase rollover current [1,2,3]. We simultaneously achieve high bandwidth (20–25 GHz), high power conversion efficiency (~30%), and relatively high (for only three VCSELs) optical output power (20–50 mW) as part of our quest to develop a key enabling component for short-distance (meters to tens of meters) fifth generation (5G) and sixth generation (6G) optical wireless (free-space) communication networks and sensing systems.

We present our motivation and a brief history of VCSEL arrays designed specifically for free-space optical communication. We disclose a new epitaxial VCSEL layer design with a bottom distributed Bragg reflector that includes AlAs (binary) layers. We next describe our triple VCSEL array geometry and our on-wafer device testing methods. Finally, we present new on-wafer measured results, briefly analyze the results, and, for the first time, compare our measured emission spectra to simulated cold cavity two-dimensional (2D) near-field surface mode wavelengths.

### 1.1. Motivation

State-of-the-art fourth generation (4G) mobile networks employ the long-term evolution (4G-LTE) standard and operate at up to 100 megabits per second (Mb/s), although the average downloading data rate is only 33.88 Mb/s [4]. These networks enable high-definition (HD) multimedia communication such as streaming high-definition movies and online gaming. The new 5G mobile wireless infrastructure (now in the early deployment stage) promises to add a plethora of ubiquitous and novel communication and sensing devices and systems to existing networks, enabling massive scales of data generation, data transfer, and data storage with anticipated data rates of 5 Gb/s to possibly 20 Gb/s via millimeter wave transmission.

The 5G era promises extreme mobile coverage, mixed virtual reality, and low-latency (aiming at <1 ms) transfers, most especially in urban and/or crowded areas. The 5G millimeter wave networks offer drastically faster bit rates (compared to the low to mid band spectrum used for 4G) but require massive numbers of antennas in each 5G base station to dynamically focus the emitted beam toward a particular receiving device (using a technique called beamforming) such as a laptop, smartphone, Internet of Things node, or autonomous vehicle. This is because millimeter waves do not penetrate walls and are commonly disrupted by interfering objects [5]. Similarly, potentially easy-to-set-up and fast-to-reconfigure VCSEL-based free-space optical communication links require beam shaping and line-of-sight transmission and, thus, are well suited to enhance (operate in parallel as a second source) or replace 5G millimeter wave links in backhaul (using the same towers), pole-to-home, and small cell applications.

The developers of future 6G networks foresee operating at terabit per second data rates by circa the 2030s, thus enabling fully immersive environments and experiences that fuse (or completely blur) cyberspace and real space instantaneously via an Internet of Everything, and thus providing complete coverage (air, space, sea, land, ultra-precise object positioning and tracking, situational awareness, etc.) via overlapped dynamic network topologies managed by artificially intelligent agents or similar machine learning control systems [6]. We seek to add to the 5G and 6G ideas [7,8,9] the possibility of flexible, rapidly deployed, free-space line-of-sight optical communication links (e.g., secure and eye safe backhaul links, personal (in home) networks, data center links, mesh networks, and much more) based on energy-efficient infrared data and sensing beams produced by low-cost and energy-efficient (low element count) VCSEL arrays.

### 1.2. VCSEL Arrays for Free-Space Optical Communication

The use of VCSELs for data communication across optical fiber is well established, as is the use of VCSEL arrays for various optical sensing and high optical output power (directed energy) applications. In contrast, the use of VCSELs for free-space optical communication is largely unexplored. There are only a few published free-space optical communication link experiments to date [10,11], wherein the authors employed a commercially available VCSEL or VCSEL array. Standards (other than for laser eye safety) for VCSEL-based free-space optical communication that specify the allowable emission wavelength or wavelengths, beam shape, minimum received power, modulation scheme, or bit error rate do not yet exist [12].

There are only a few research papers to date specifically on the development of VCSEL arrays for free-space optical links. Our experimental arrays trace their origins to the pioneering work of Yoshikawa et al., in 2005 [13], wherein the authors demonstrated 3 × 3, 4 × 4, and 5 × 5 850 nm VCSEL arrays emitting cone-shaped beams at 2.5 Gb/s, intended specifically as light sources for free-space data links. In 2009 and 2010, Safaisini et al. [14,15] reported 980 nm 28-element arrays with a bandwidth (*f*_3dB_) of 7.6 GHz, maximum wall plug efficiency (WPE_max_) of ~12%, and maximum optical output power of *L*_max_ ~150 mW. In 2020, Khan et al. reported a 940 nm 3 × 3 VCSEL array with *f*_3dB_ = 10 GHz, WPE_max_ = 20%, and *L*_max_ = 62.4 mW [16]. Otherwise, the only other work (to our knowledge) on VCSEL arrays specifically for future free-space optical communication is our recent work on small (3–19-element) VCSEL arrays [17,18,19,20].

In [18], we reported the static and dynamic characteristics of a three-element electrically parallel, optically uncoupled 980 nm VCSEL array with *ϕ*~7.5 μm. We achieved a maximum *f*_3dB_ of ~24 GHz, WPE_max_ of ~36%, and *L*_max_ of ~27 mW. In this new work, we characterize a binary DBR version of the same triple-array geometry but over a much larger range of oxide aperture diameters, from *ϕ*~6 μm to 17 μm. In a new key plot of *f*_3dB_ versus *L* (with *ϕ*~6, 9, and 14 μm), we illustrate the possible bandwidth–optical output power combinations of our three-element electrically parallel arrays.

## 2. Materials and Methods

We disclose in three subsections our VCSEL epitaxial material layer design, our three-element VCSEL array physical geometry and fabrication methods, and finally our on-wafer VCSEL testing methods.

### 2.1. VCSEL Epitaxial Design

**Figure 1:**
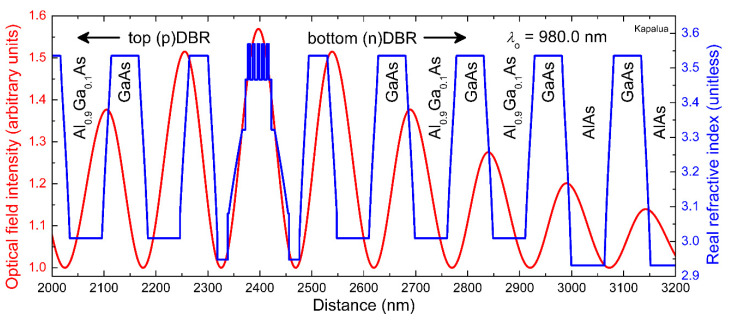
Simulated one-dimensional optical field intensity on resonance (red curve) and real refractive index (blue curve) along the epitaxial growth direction in and around the quantum well (QW) active region for the Kapalua 980 nm vertical cavity surface emitting laser (VCSEL) design.

We grew 980 nm VCSEL material layers on highly n-doped (001) surface-oriented GaAs substrates via metal–organic vapor phase epitaxy (MOVPE)—in batches of 12, 3 inch wafers per growth run. For the particular wafer set reported herein—our *Kapalua* VCSEL epitaxial design—we started with 200 nm of undoped (u)GaAs; then, in succession, we grew 5 nm of graded (u)Al_x_Ga_1−x_As (with *x* varying from 0.0 to 1.0), 157 nm of (u)AlAs, 10 nm of graded (u)Al_x_Ga_1−x_As (*x* = 1.0 to 0.0), and 1580 nm of GaAs highly n-doped with silicon. This thick (n+)GaAs layer served as an n-metal (cathode) ohmic contact layer. The VCSEL epitaxial structure followed, including in sequence, a 34-period n-doped AlAs/GaAs distributed Bragg reflector (DBR) with 18 nm thick graded interfaces (from an AlAs mole fraction *x* = 1.0 to 0.0 and vice versa), a three-period (n)Al_0.9_Ga_0.1_As/GaAs DBR with 18 nm thick graded interfaces (from *x* = 0.9 to 0.0 and vice versa), a λ/2 optically thick cavity, and a 15.5-period p-doped with carbon top coupling (p)Al_0.9_Ga_0.1_As /GaAs DBR (with the same 18 nm thick graded interfaces). We employed five In_y_Ga_1−y_As (*y* ~0.23) quantum wells (QWs) surrounded by GaAs_1−x_P_x_ (*x* ~0.14) barrier layers and two, 20 nm thick (as grown) Al_0.98_Ga_0.02_As layers, selectively thermally oxidized during device fabrication, centered on optical field intensity nodes, as shown in Figure 1.

### 2.2. VCSEL Array Geometry and Fabrication

**Figure 2:**
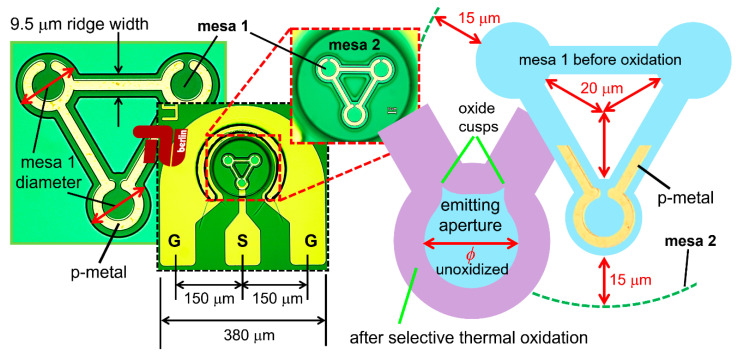
Illustration of the triple VCSEL array geometry via a collage of microscope images and schematics. We show unoxidized areas in light blue and oxidized areas in purple.

We fabricated VCSELs on quarter wafer pieces in our university cleanroom and thermal oxidation laboratory; see Figure 2 for images and illustrations of our three-element (triple) VCSEL array device geometry. We deposited ZnAu/Au p-metal (with cut open rings plus inter-ring metal lines) via thermal evaporation, and then etched circular top mesas just through the optical cavity with Cl_2_ + BCl_3_ in a SENTECH Instruments GmbH SI 500 (Berlin, Germany) inductively coupled plasma (ICP) reactive ion etching (RIE) system using photoresist as an etching mask. We monitored the etch depth in situ (during the ICP-RIE etching) by measuring the optical power reflectance of several wafer surface points at *λ*_o_ = 632.8 nm. After performing a selective thermal oxidation at 420 °C at 50 mbar in a chamber filled with N_2_ + H_2_O vapor, we etched a second circular mesa down just into the 1580 nm thick (n+)GaAs and then deposited Ni/AuGe/Au n-metal (cathode) in horseshoe shapes around the bottom mesas. Next, we spun-on and pre-baked photosensitive benzocyclobutene (BCB) to help planarize the arrays (to create a quasi-flat surface for subsequent pad metal deposition), and then exposed the BCB to ultraviolet (UV) radiation and developed (removed) BCB above the p-metal and n-metal. Lastly, we deposited Cr/Pt/Au contact metal via standard UV contact photolithography in a lift-off process in the shape of co-planar ground–signal–ground (GSG) pads to facilitate on-wafer probing. The linear spacing of the GSG pads (the center of the S pad to the centers of each G pad) was 150 μm. Our co-planar GSG pad configuration had a fixed width of 480 μm (as indicated in Figure 2) and heights that varied from roughly 350 (Row 0) to 370 μm (Row 9). To facilitate on-wafer testing, we placed our triple arrays within the GSG pad configuration shown in Figure 2, i.e., the same contact pad geometry we used for single VCSELs.

We constructed electrically parallel, optically uncoupled triple (three-element) 980 nm VCSEL arrays with variable oxide aperture diameters and variable inter-VCSEL pitch (separation distance) as follows [17,18]. The top mesas (mesa 1) consisted of three circles with identical diameters, each centered on one of the three imaginary vertices of an equilateral triangle. The mesa 1 diameters ranged from 22 μm (Row 0) to 31 μm (Row 9). We connected the three top mesa circles with 9.5 μm wide (rectangular) ridge connectors. Each top mesa, thus, had a distinct open triangular pattern with solid circular corners. We centered 3.5 μm wide p-metal contacts on our top mesas, but with ring patterns on the three corners. We electrically isolated the ridge connectors via selective thermal oxidation; thus, we oxidized a minimum length of 9.5/2 μm in from the exposed mesa 1 edges after the first dry etch, and simultaneously formed circular oxide apertures within the metal rings (possibly with negligible cusps as shown in Figure 2). The inter-VCSEL spacing (VCSEL center to VCSEL center) varied from about 54 μm (Row 0) to 62 μm (Row 9). The three VCSELs (in each array) shared a circular bottom mesa (mesa 2) with diameters ranging from 114 μm (Row 0) to 132 μm (Row 9). The radial distance from the centers of the bottom mesas to the nearest inner edges of the top mesas was constant at 20 μm for all triple arrays (Rows 0 to 9). Likewise (see the dimensions in Figure 2), the radial distance from the outer edges of the top mesas to the edges of bottom mesas was constant at 15 μm for all triple arrays (Rows 0 to 9).

### 2.3. VCSEL Charcaterization Methods

We measured the triple VCSEL array characteristics using the methods and test equipment we described in [17,18,19,20]. Herein, we performed all measurements at ambient room temperature (RT ~23 °C) on three separate test stations in our university laboratory. We placed our fabricated quarter wafer pieces (one at a time) on a first probe station platen (a flat metal surface with several small vacuum holes), holding the wafer in place via a vacuum but otherwise without heat sinking. By means of high-frequency GSG probes, we directly contacted the VCSEL array under test. Via our proprietary LabVIEW (computer) program, we swept the bias current (*I*) with a Keithley 2400-LV current source and measured the static light output power (*L*) and voltage (*V*), thus producing *LIV* curves. We measured *L* with a calibrated integrating sphere which included a photodiode (PD, with a wavelength sensitivity range well below and above 980 nm). With another Keithley 2400-LV, we measured the current from the integrating sphere plus PD and converted the current to an optical power. We placed a 90% blocking neutral density filter (with antireflecting coatings at 980 nm) between our VCSEL under test and the entrance to the integrating sphere to limit the input optical power. On a second probe station, we placed a GSG probe onto the VCSEL array and applied a static (direct current, DC) as before via a LabVIEW program (either a fixed bias current or a series of bias currents). We placed a cleaved-end OM1 multiple-mode optical fiber (MMF) directly onto the three VCSELs (butt-coupled). We connected the opposing end of the OM1 MMF patch cord (typically 0.5 or 1 m in length) to an Ando model AQ 6317C optical spectrum analyzer (Yokogawa Electric Company Limited, Hiroshima, Japan) and recorded the emission spectra.

On the same second probe station, we performed small-signal frequency response (i.e., S21 scattering parameter) measurements under the control of another LabVIEW program via standard daisy-chained general-purpose interface bus (GPIB) cables connected to the test equipment. We connected port 2 of an Hewlett-Packard 8722C (Palo Alto, CA, USA) Vector Network Analyzer (VNA) to our VCSEL via a high-frequency metal line and GSG probe (which electrically excited the VCSEL array with a direct current (DC) bias (from a Keithley 2400-LV current source) plus a sinusoidal −20 dBm (10 μW) source signal created by the VNA. We butt-coupled the same OM1 MMF (used in our spectral emission measurement) to the VCSEL array under test and connected the opposing end to a New Focus (San Jose, CA, USA) model 1424 photodetector with a bandwidth of 25 GHz. The New Focus photodetector was connected directly to the VNA’s port 1. We calibrated the VNA test setup to mitigate errors prior to our S21 measurements. Note that we (actually) measured the S12 parameters since we connected the VNA’s port 2 (electrically) to the VCSEL via a high-frequency (HF) transmission line and we connected the photoreceiver (electrically) to the VNA’s port 1. Since the VNA was a two-port two-path VNA, the port connections were interchangeable. This was simply because port 2 was closer to our probe station and VCSEL under test. The result was identical if we switched the port connections. By convention, we report the frequency response as S21 parameters.

Lastly, for data transmission measurements using two-level pulse amplitude modulation at 25 to 40 Gb/s (see [18] for a schematic of the test setup), we placed the fabricated wafers onto a third test station and made electrical contact with a GSG probe on one array at a time. Via an SHF 12100B (Berlin, Germany) bit pattern generator (which received a clocking signal from an Agilent E8257D (Santa Clara, CA, USA) signal generator) and a bias-tee, we excited the VCSEL array with a DC bias current plus (as a large signal peak-to-peak voltage (typically ~610 mV) on top of the DC bias), a standard pseudorandom bit (or binary) sequence (PRBS) of word length 2^7^ − 1.

Via an OM1 MMF patch cord (~1 m in length), we coupled the array emission into a highly sensitive u2t (Berlin, Germany) photoreceiver (a custom-built PD and amplifier with a bandwidth of 30 GHz), and, in turn, we sent the resultant electrical signal into either an Agilent DCA J86100C (Santa Rosa, CA, USA) digitizing oscilloscope (to measure eye patterns) or into an SHF 11100B (Berlin, Germany) error analyzer (ER) to measure the bit error ratio. We first optimized the eye pattern at 40 Gb/s by adjusting the bias current, the magnitude of the peak-to-peak large signal PRBS signal, and, if necessary, the position of the OM1 MMF. We then switched the OM1 MMF from the digitizing oscilloscope to the error analyzer, and, while measuring the bit error ratio (BER) in real time, we tweaked the voltage detection level (within the eye pattern) to optimize the BER. Then, we proceeded with the actual BER test measurements. First, without optical attenuation, we ran the PRBS for 30 s or longer and recorded the BER. We measured the optical power entering the u2t photoreceiver with a JDSU OLP-55 (Milpitas, CA, USA) optical power meter. We then repeatedly reconnected the fiber to the u2t photoreceiver, added ever higher optical attenuation (to add errors) using a JDSU OLA-54 variable optical attenuator, and repeated the BER measurement.

## 3. Results

We performed all measurements at room temperature (the ambient *T* was ~23 °C) in our university laser diode testing laboratory without heat sinking via on-wafer probing. Herein, we present results from two fabricated quarter wafer pieces (from a 3 inch diameter starting wafer), designated Kapalua NE (which we thermally selectively oxidized for 111 min) and Kapalua SE (which we thermally selectively oxidized for 135 min). Each processed quarter wafer contained roughly eight unit cells (UCs). Each repeated unit cell contained 184 VCSELs (single VCSELs, triple VCSEL arrays, septuple VCSEL arrays, and more). We focus herein solely on selected columns of triple VCSELs (groups of 10 adjacent triple arrays in Rows 0 to 9 in each unit cell). The triple VCSEL arrays on Kapalua NE had oxide aperture diameters ranging from ~8 to 17 μm (Rows 0 to 9) with corresponding top mesa diameters of 22 to 31 μm, whereas the triple VCSEL arrays on Kapalua SE had oxide aperture diameters ranging from ~5 to 14 μm (Rows 0 to 9) with the same top mesa (mesa 1) diameters.

### 3.1. Static Optical Output Power–Current–Voltage Characteristitcs

**Figure 3:**
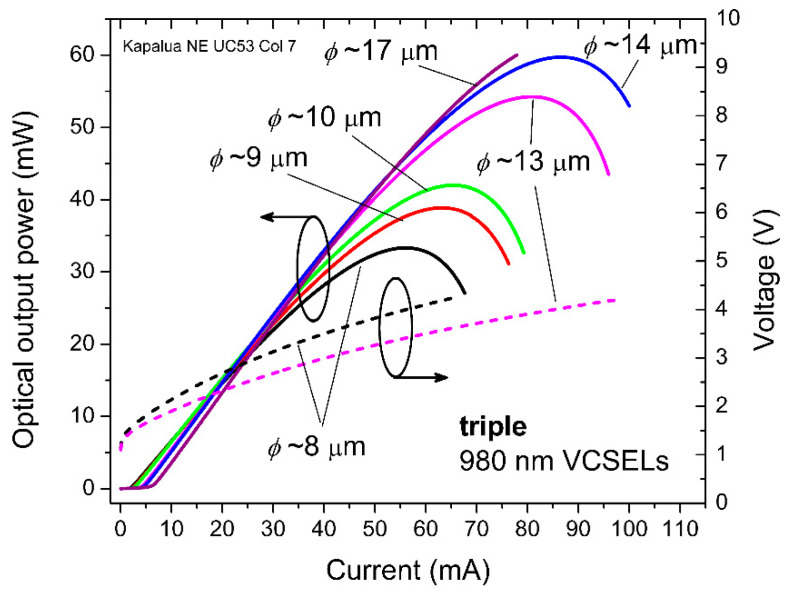
Measured static optical output power and voltage as functions of applied direct current (DC) bias current for 980 nm triple VCSEL arrays with oxide aperture diameters (*ϕ*) from ~8 to 17 μm.

In Figure 3, we plot example static (direct current, DC) *LIV* curves for triple 980 nm VCSEL arrays with variable oxide aperture diameters from (estimated) *ϕ*~8 to 17 μm. The maximum *L* (at the *LI* rollover points) vary from about 30 mW (or ~10 mW per *ϕ*~8 μm VCSEL) to more than 60 mW (or ~20 mW per *ϕ*~17 μm VCSEL). The triple VCSEL array (on the NE quarter wafer) with *ϕ*~8 μm lies in Row 0 with a top mesa 1 diameter of 22 μm. The triple VCSEL with *ϕ*~17 μm lies in Row 9 with a top mesa 1 diameter of 31 μm.

**Figure 4:**
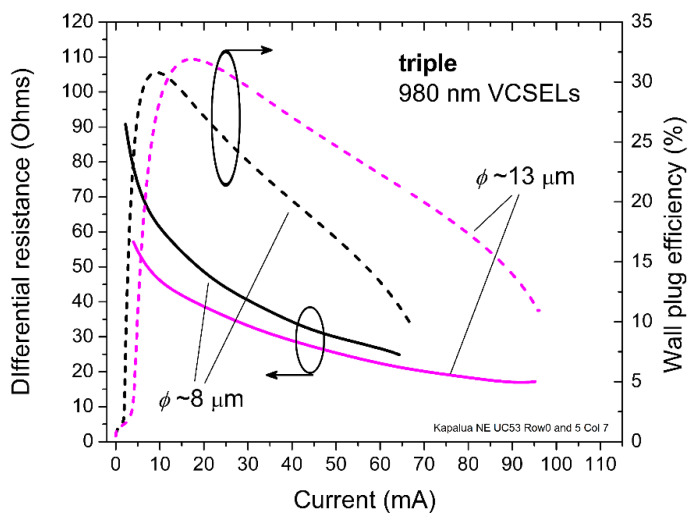
Extracted differential series resistance and wall plug efficiency versus DC bias current for the 980 nm triple VCSEL arrays in Figure 3 with *ϕ*~8 and 13 μm.

In Figure 4, we plot the corresponding differential series resistance (*R*_diff_ = *ΔV/ΔI* in Ω) and the wall plug efficiency (WPE = 100·*L*/(*I·V)* as a percentage), both as functions of the applied forward DC bias current for the triple arrays with *ϕ*~8 μm and *ϕ*~13 μm. For the *ϕ*~8 μm triple array, the maximum WPE is about 30% near *I* = 10 mA, while *R*_diff_ drops from roughly 70 Ω at *I* = 10 mA to roughly 30 Ω at *I* = 50 mA. For the *ϕ*~13 μm triple array, the maximum WPE is about 32% near *I* = 15 mA, while *R*_diff_ drops from roughly 45 Ω at *I* = 10 mA to roughly 30 Ω at *I* = 45 mA. Since the three VCSELs are electrically in parallel, *R*_diff_ is one-third the differential series resistance of one of the three VCSELs (neglecting any resistance differences among the three VCSELs due to the array geometry).

**Figure 5:**
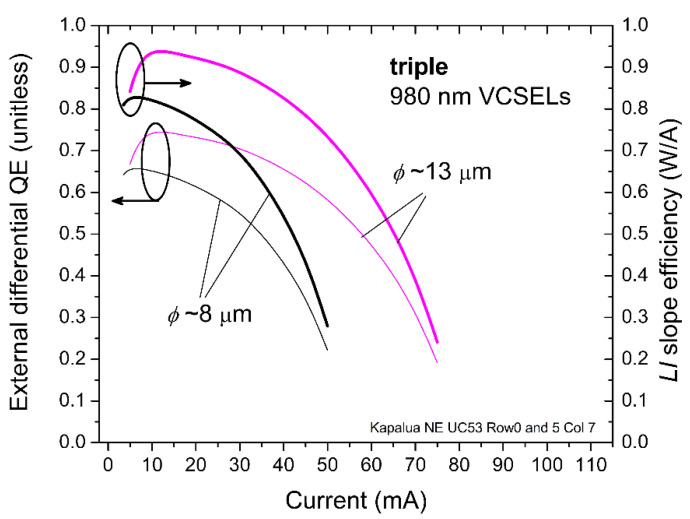
Extracted external differential quantum efficiency (QE) and *LI* slope efficiency versus DC bias current for the 980 nm triple VCSEL arrays in Figure 3 with *ϕ*~8 and 13 μm.

Next, in Figure 5, we plot the unitless external differential quantum efficiency *η*_QE_ *= (qλ*_o_*/hc)(ΔL/ΔI)* versus *I*, where *q* is the charge on one electron, *h* is Planck’s constant, *λ*_o_ is the emission wavelength (980 nm), and *c* is the speed of light. We also plot, in Figure 5, the *LI* slope *η*_LI_ *=* *ΔL/ΔI* (in W/A) for the triple arrays with *ϕ*~8 μm and *ϕ*~13 μm. For the 13 μm triple VCSEL array, *η*_QE_ and *η*_LI_ peak at ~0.7 (unitless) and ~0.9 (W/A) at about *I* = 10 mA and drop to about half these magnitudes at *I* ~70 mA. For the 8 μm triple VCSEL array, *η*_QE_ and *η*_LI_ peak at ~0.65 (unitless) and ~0.82 (W/A) at about *I* = 5 mA and drop to about half these magnitudes at *I* ~45–50 mA.

### 3.2. Emission Spectra and 2D Optical Mode Modeling

**Figure 6:**
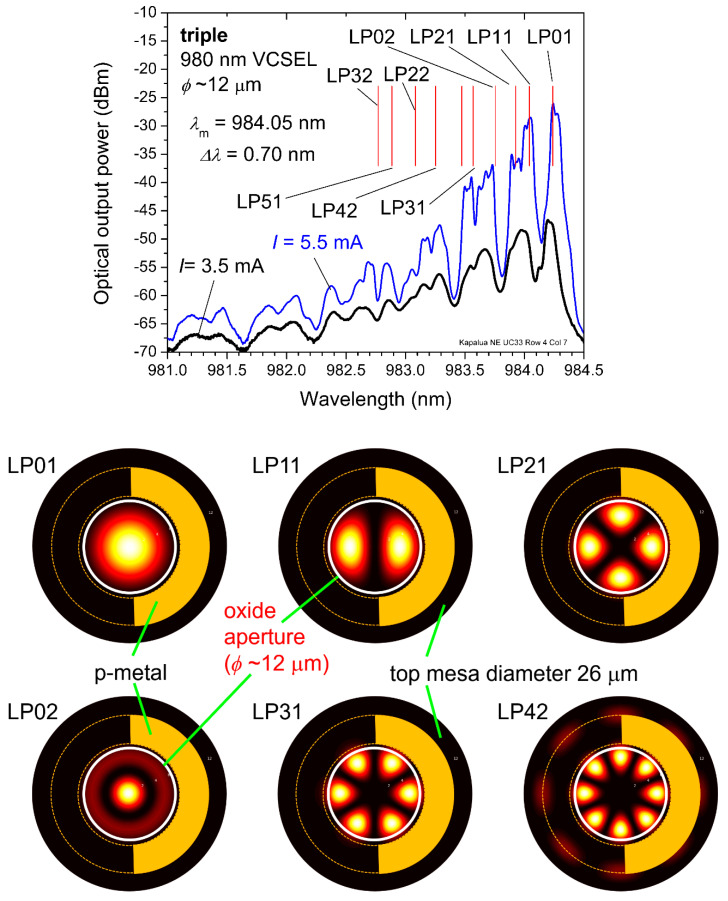
(**top**) Measured spectral emission for a 980 nm triple VCSEL array with *ϕ*~12 μm and top mesa diameters of 26 μm, at static bias currents of *I* = 3.5 mA (below threshold) and *I* = 5.5 mA (above threshold). The vertical red lines indicate the wavelengths of some of the simulated LP modes. (**bottom**) Simulated (cold cavity) two-dimensional (2D) near field (surface) modes (normalized resonant optical field intensities) of any one VCSEL in the array. The array includes 3.5 μm wide (and 3 μm from the top mesa 1 edge) top surface p-metal contacts, partially removed here for ease of viewing (but included in the simulations). In the simulation, we adjusted the VCSEL etalon to fit the LP01 mode wavelength at *I* = 3.5 mA; all other wavelengths followed naturally.

In Figure 6 (top), we plot the measured emission spectra of a triple 980 nm VCSEL with *ϕ*~12 μm at a DC bias *I* = 3.5 mA (below threshold) and at *I* = 5.5 mA (above threshold). A common figure of merit for emission spectra (measured as a series of discrete points) is the root-mean-square spectral width (in nm), which is calculated as follows [21,22,23]:

(1) $$\Delta \lambda =\sqrt{\sum_{i=1}^{n}{\left(\lambda_{i}-\lambda_{m}\right)}^{2}}\;\;\mathrm{where}\;\lambda_{m}=\sum_{i=1}^{n}\frac{P_{i}}{P_{tot}}\lambda_{i},\;\;\mathrm{and}\;P_{tot}=\sum_{i=1}^{n}P_{i},$$

where *n* is the number of discrete measurement points, *P_i_* is the optical power (in mW) at point *i*, *λ_i_* is the wavelength at point *i* (in nm), and *λ_m_* (in nm) is the mean (average) wavelength. When *I* = 5.5 mA, we get *λ_m_* = 984.05 nm and *Δλ* = 0.70 nm.

**Figure 7:**
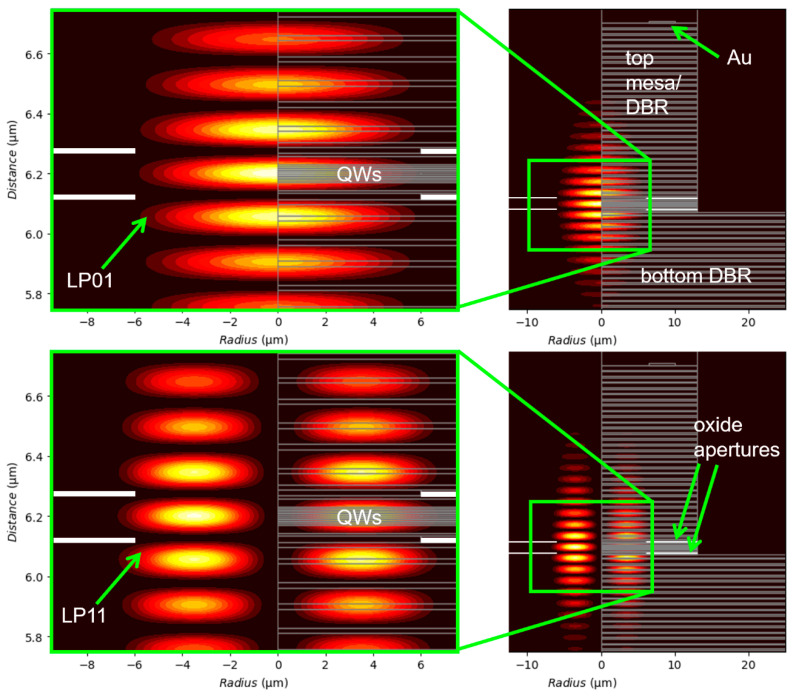
Simulated two-dimensional LP01 and LP11 cold cavity modes (normalized resonant optical field intensities) for a cylindrically symmetric (rotated 2π about the vertical line at radius = 0) 980 nm VCSEL with *ϕ*~12 μm (corresponding to any one of the three VCSELs in the triple array in Figure 6).

We performed two-dimensional cold cavity mode simulations via the effective frequency method [24] as implemented in [25]. We included near-field images for several example optical modes. Each allowed mode had a corresponding wavelength, which we plot as straight red lines in Figure 6 (top). For example, the LP12 mode (the only mode not labeled in Figure 6 (top)) is at *λ*_o_ = 983.47 nm. Comparing the measured emission spectra to the simulated modes, we find that (linearly polarized) modes LP01, LP11, LP21, LP02, and others match well to the measured data. Note that our cold cavity simulations did not include the impact of temperature (self-heating), spatial index of refraction variations due to injected carriers, spatial variations in QW gain, and more. In Figure 6 (bottom), we plot the corresponding near field (surface) patterns for the LP01 and LP11 modes and for the simulated LP21, LP02, LP31, and LP42 modes. In Figure 7, we plot two-dimensional (2D) cross-sections of the simulated LP01 and LP11 modes. In future work, we plan to compare measured and simulated near-field surface patterns.

As a sanity check, we used the equation in [26] to estimate (to first order) the *ϕ* for the 12 μm triple VCSEL as follows. The estimated mode size (for a square aperture of side length *b* which we took as *ϕ* ≈ *b* is $b\approx sqrt\left\{\left(3\pi^{2}/n_{ave}^{2}\right)\cdot \left(1/\left[{\left(2\pi /\lambda_{1}\right)}^{2}-{\left(2\pi /\lambda_{0}\right)}^{2}\right]\right)\right\}$. In Figure 6 (top), the fundamental mode peak *λ*_o_ = 984.208 nm and the next higher-order mode peak *λ*_1_ = 983.980 nm when *I* = 3.5 mA. With the average optical cavity refractive index $n_{ave}\;$≈ 3.3, we find *ϕ*≈11,997 nm (~12 μm). As a third method to estimate *ϕ*, we performed selective thermal oxidation tests on several Kapalua test pieces as described in [17] to determine the relationship between oxidation length and oxidation time. From these data and our knowledge of the top mesa diameters, we estimated our *ϕ*.

### 3.3. Small-Signal Frequency Response

**Figure 8:**
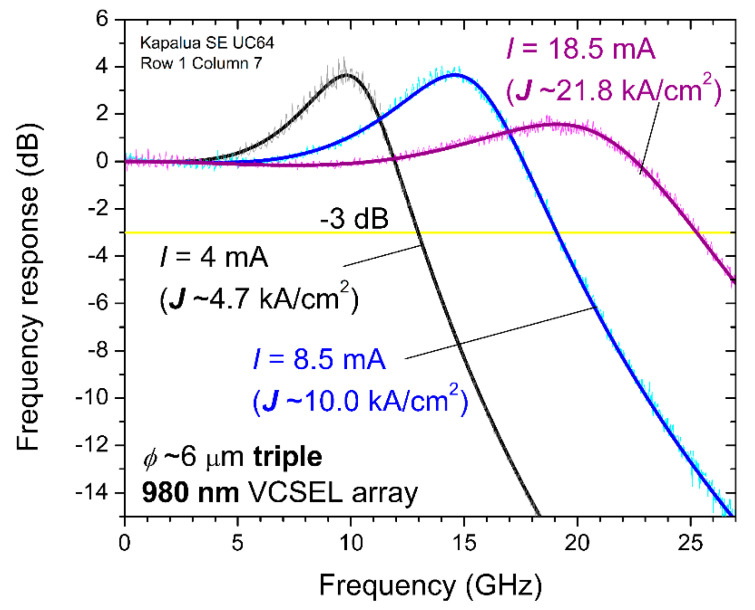
Measured small-signal modulation frequency response (|S21|) for a 980 nm triple VCSEL array with *ϕ*~6 μm at direct current (DC) bias currents *I* = 4, 8.5, and 18.5 mA.

We performed small-signal modulation frequency response measurements primarily to determine the −3 dB bandwidth of our triple VCSEL arrays. As an example, in Figure 8, we plot, after correcting the data for the frequency response of the New Focus 25 GHz bandwidth photoreceiver, the measured |S21| (absolute value magnitudes) versus frequency of a 980 nm triple VCSEL array with *ϕ*~6 μm at DC bias currents of *I* = 4, 8.5, and 18.5 mA (corresponding to bias current densities of about 4.7, 10.0, and 21.8 kA/cm^2^). We fit the measured |S21| data to the following equation [18]:

(2) $$\left|S21\right|=c\;+20\cdot log_{10}\left(\sqrt{\frac{f_{R}^{4}}{{\left(f_{R}^{2}-f^{2}\right)}^{2}+{\left(\gamma f/2\pi \right)}^{2}}\cdot \;\frac{1}{1+{\left(f/f_{p}\right)}^{2}}}\right)$$

where *c* (in dB) is a constant we use to set the fitted frequency response to the reference level 0 dB at the frequency *f* = 0 Hz, *f_R_* is the relaxation oscillation resonance frequency (in GHz), *γ* is the damping (in GHz or equivalently in ns), and *f_p_* is the parasitic frequency (in GHz). In Table 1, we list the fitting parameters and measured bandwidths for the 6 μm triple VCSEL array corresponding to the measured data in Figure 8.

**Figure 9:**
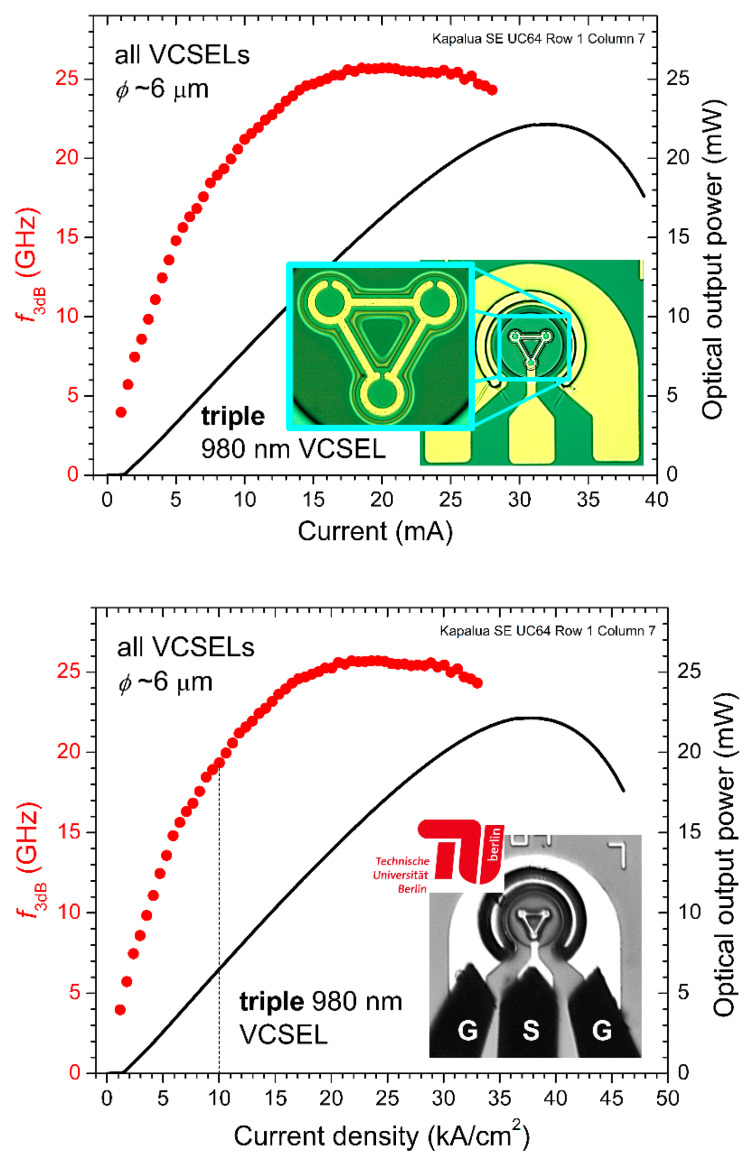
Measured −3 dB small-signal modulation bandwidth (*f*_3dB_) versus direct current (DC) bias *I* (upper plot) and versus ***J*** (lower plot) for a 980 nm triple VCSEL array with *ϕ*~6 μm.

From the |S21| data, we found the −3 dB small-signal modulation bandwidths (*f*_3dB_) for the triple array—i.e., the frequency *f* when |S21| = −3 dB in Equation (2). In Figure 9, we plot *f*_3dB_ as a function of DC bias current (top plot) and as a function of DC bias current density (bottom plot) for the 6 μm triple VCSEL array. The maximum bandwidth is just above 25 GHz for *I* ~18 to 20 mA, a record value for our triple VCSEL arrays.

**Figure 10:**
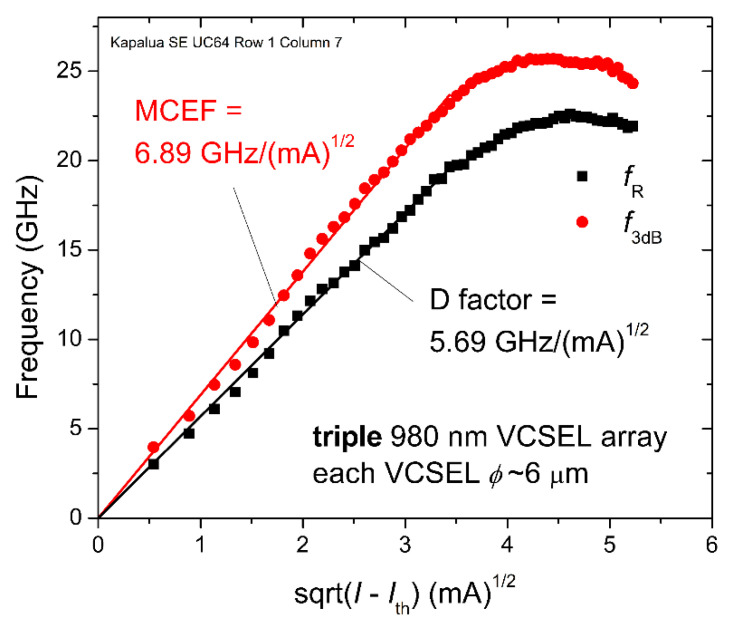
Measured −3 dB bandwidth (*f*_3dB_) and relaxation oscillation resonance frequency (*f_R_*) versus (*I* − *I*_th_)^1/2^ for the 980 nm triple VCSEL array with *ϕ*~6 μm in Figure 9.

Next, in Figure 10, we plot *f_R_* and *f*_3dB_ versus (*I* − *I*_th_)^1/2^ and extract two common dynamic VCSEL metrics: the D factor given by *f_R_* = *D*·(*I* − *I*_th_)^1/2^ and the modulation current efficiency factor MCEF given by *f*_3dB_ = MCEF·(*I* − *I*_th_)^1/2^, where *I*_th_ is the threshold current. Thus, *D* = 5.69 GHz/(mA)^1/2^ and MCEF = 6.89 GHz/(mA)^1/2^ are the linear slopes in Figure 10 at the lower values of *I*.

### 3.4. Data Transmission

To demonstrate the potential of our triple arrays as sources for free-space optical communication, we performed large signal modulation data transmission experiments at room temperature on a triple VCSEL array with *ϕ*~6 μm, but across an OM1 multiple-mode fiber (MMF) patch cord (as we are not equipped for free-space measurements) as described in Section 2 via two-level pulse amplitude modulation (PAM-2) with a pseudorandom binary sequence (PRBS) of word length 2^7^ − 1. With butt-coupling of the OM1 MMF (with a core diameter of 62.5 μm) directly on the *ϕ*~6 μm triple VCSEL array (where the center of each VCSEL emitting aperture lies 31.5 μm from the triple VCSEL centroid), we coupled at most ~44% of the array emission from each VCSEL into the fiber (neglecting surface reflection). For 100% coupling, we would need one or more lenses to focus the emission into the optical fiber. For free-space transmission across a room or across a street, we would similarly require a series of lenses. For a practical free-space optical communication system, we would seek purposely to detect only a fraction of the optical intensity in a given beam (of data), especially if we employ a conical beam to cover a large detection area with multiple photoreceivers.

**Figure 11:**
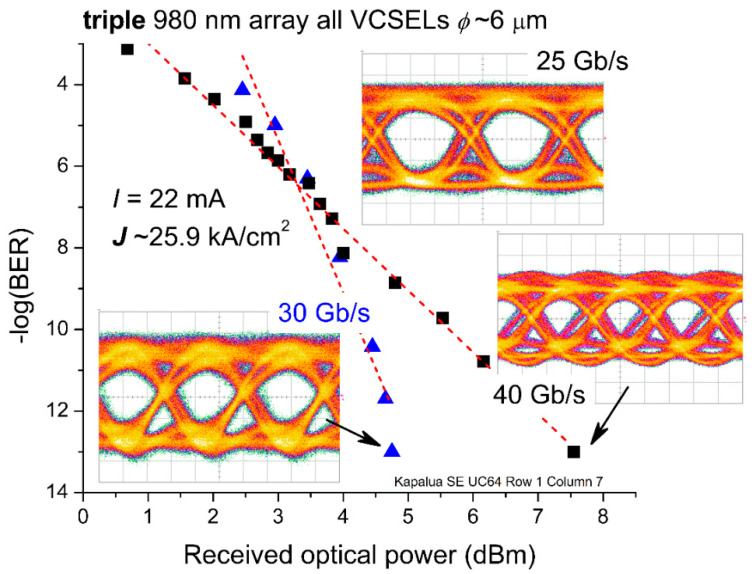
Bit error ratio (BER) versus received optical power for a 980 nm triple VCSEL array with *ϕ*~6 μm at 30 and 40 Gb/s, via a back-to-back data transmission test across OM1 multiple-mode fiber (MMF) with two-level pulse amplitude modulation (PAM-2, nonreturn to zero) and a pseudorandom binary sequence (PRBS) of word length 2^7^ − 1. We optimized the BER test settings for 40 Gb/s and used the same settings at 30 Gb/s (thus leading to the smaller received optical power at 40 Gb/s when the BER exceeds 1 ×10^−6^). Inset images: eye patterns at 25, 30, and 40 Gb/s taken at the minimum BER (with zero added optical attenuation).

In Figure 11, we plot the negative logarithm (base 10) of the bit error ratio (BER) versus the received optical power for a DC bias *I* = 22 mA (***J***~25.9 kA/cm^2^) for bit rates of 30 and 40 Gb/s. We first optimized the eye pattern for 40 Gb/s, recorded the 40 Gb/s and 30 Gb/s eye patterns with zero attenuation (the inset images in Figure 11), and used the same settings for the subsequent BER test at 30 Gb/s. Interestingly, since we optimized the testing for 40 Gb/s, the received optical power for the 40 Gb/s data was smaller compared to that for the 30 Gb/s data when the −log_10_(BER) was below 6. We recorded zero errors when we set the optical attenuation to 0 dB (over a period exceeding 30 s); therefore, we set the BER to 1 × 10^−13^. At 40 Gb/s, we transmitted ≥1.2 × 10^12^ bits (≥9.3 billion PRB sequences), and then recorded the BER. As a check, we repeated the BER tests over 2 h testing periods and found the resulting BER with zero attenuation unchanged.

## 4. Discussion

**Figure 12:**
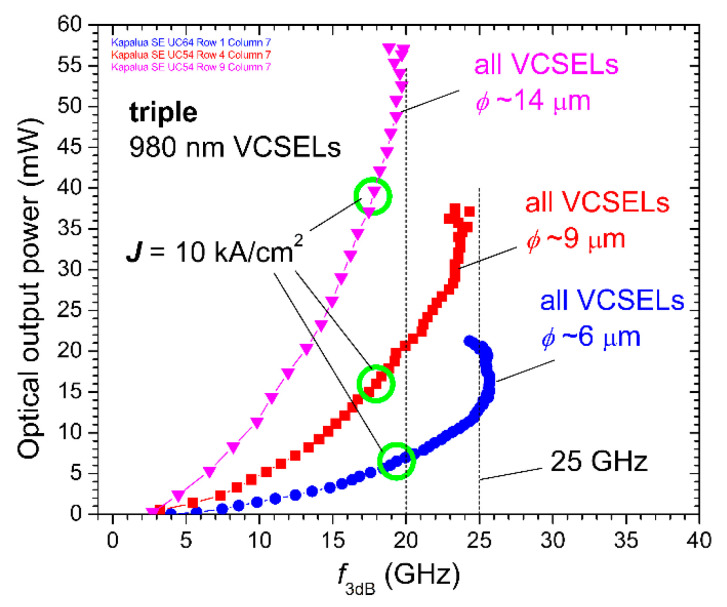
Extracted *f*_3dB_ versus static optical output power (*L*) for 980 nm triple VCSEL arrays with *ϕ*~6 μm (blue circles), *ϕ*~9 μm (red squares), and *ϕ*~14 μm (magenta triangles). The green circles indicate the points where the bias current density ***J*** = 10 kA/cm^2^.

Our 980 nm triple VCSEL arrays exhibited record combinations of bandwidth, optical output power, and efficiency (WPE, *η*_LI_, and *η*_QE_); however, when are arrays a superior choice compared with single VCSELs for optical wireless communication or sensing applications? Consider the data in Figure 12, wherein we plot the −3 dB small-signal modulation bandwidth *f*_3dB_ versus the static (continuous wave) optical output power *L* for triple 980 nm VCSEL arrays with *ϕ*~6, 9, and 14 μm. We generated the plot by matching our measured *f*_3dB_ versus *I* data (as in Figure 9 (top)) to our static *L* versus *I* data. From the curves, we deduced that the maximum bandwidth of the 9 μm array (*f*_3dBmax_~24 GHz) is only slightly lower than the *f*_3dBmax_ (~26 GHz) for the 6 μm array, but the optical output power of the 9 μm array at *f*_3dBmax_ (*L*~35 mW) is more than double the optical output power (*L*~15 mW) at *f*_3dBmax_ for the 6 μm array. We may increase *L* to ~50 mW with our 14 μm array, but with a reduced *f*_3dBmax_ of about 20 GHz.

**Figure 13:**
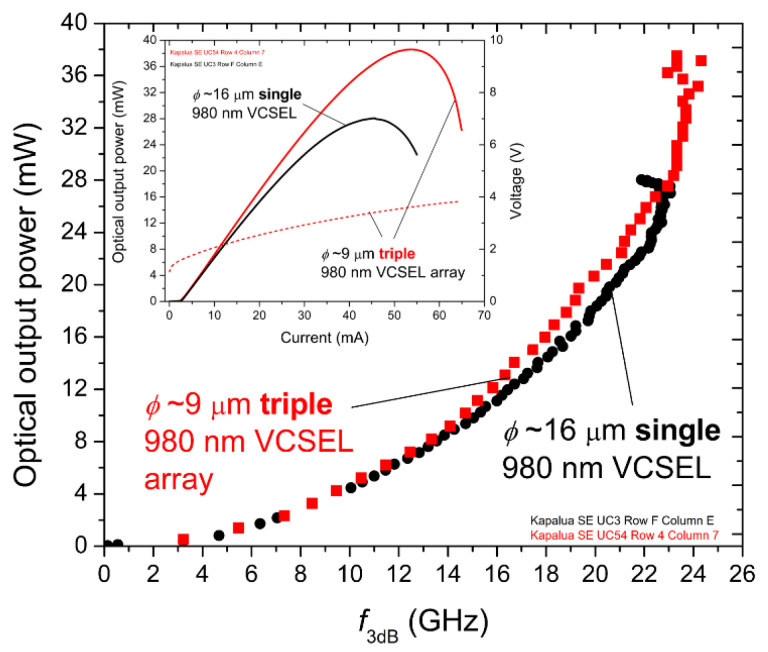
Extracted bandwidth *f*_3dB_ versus static optical output power (*L*) for a reference 980 nm single VCSEL with *ϕ*~16 μm (black circles) and for a 980 nm triple VCSEL array with *ϕ*~9 μm (red squares). Inset: the corresponding *LIV* curves. The emitting areas of the single VCSEL and the triple VCSEL array are approximately equal. The maximum *f*_3dB_ for the single *ϕ*~16 μm VCSEL is ~23 GHz. The maximum *f*_3dB_ for the triple *ϕ*~9 μm VCSEL array is ~24 GHz.

To be conservative (i.e., for reliable oxide aperture VCSEL operation), we could choose to limit the operating current density (***J***) for our arrays and our single VCSELs to ~10 kA/cm^2^ [27]. In Figure 12, we indicate the operating points where ***J***~10 kA/cm^2^ with green circles, and we find for the 6 and 9 μm arrays that the corresponding *f*_3dB_ is ~18–19 GHz but *L* is about threefold higher for the 9 μm array (~15 mW versus 5 mW) compared to *L* for the 6 μm array. As another reference point, we plot in Figure 13 *f*_3dB_ versus *L* for the same 9 μm triple VCSEL array and for a single reference VCSEL (fabricated from the same epitaxial material) with *ϕ*~16 μm.

The total emitting area of the single 16 μm VCSEL and the total emitting area of the triple 9 μm VCSEL array in Figure 13 are approximately equal (e.g., π·(16/2)^2^ ≈3·π·(9/2)^2^)—a perfect match would require *ϕ*~15.59 μm for the single VCSEL). As a result (not surprisingly), their *f*_3dB_ versus *L* data very closely overlap, although the triple array achieves a higher maximum optical output power. At the operating points where ***J***~10 kA/cm^2^, the single VCSEL and the triple array are (within experimental error) the same. A key difference (between the single VCSEL and the triple VCSEL array) not apparent in Figure 13 is the far-field pattern, which, for the large-aperture single VCSEL without lensing or engineered placement and/or interaction of the VCSEL emitters, would likely take a donut shape as typically higher-order transverse modes dominate the emission at elevated bias currents due to nonuniform current injection (i.e., current crowding near the edges of the oxide aperture layers).

## 5. Conclusions

We find that, for free-space optical communication, top-emitting electrically parallel VCSEL arrays with small to medium aperture diameters become increasingly attractive compared to larger-aperture single VCSELs as the required optical output power increases. For large numbers of elements (~570 elements as in [28] for example), we anticipate significant decreases in bandwidth for top-emitting arrays due to increased resistive and capacitive losses. By neglecting far-field patterns and beam shaping (not investigated here) at low to moderate optical output powers (about 50 mW or less), single VCSELs may suit the application. However, for medium to high power optical wireless communication and sensing, we believe that arrays are a superior choice. There remains plenty more to investigate including optimized packaging schemes, substrate emitting variations of our epitaxial designs, beam shaping/combining via novel array geometries and/or surface lenses, bandwidth–power–efficiency trade-offs for large arrays (~37 or more elements), and studies of the advantages of optically coupled and uncoupled VCSEL arrays for optical wireless communication and sensing.

Whereas herein we investigated electrically parallel, optically uncoupled triple arrays with *ϕ*~6 to 17 μm produced from our Kapalua epitaxial design, in previous work [18], we investigated a three-element 980 nm VCSEL array with *ϕ*~7.5 μm produced from our Ka’anapali epitaxial design. Our Ka’anapali epitaxial design is similar to our Kapalua epitaxial design but with 14.5 top DBR periods (one fewer than Kapalua) and with AlGaAs/GaAs bottom DBR layers (i.e., not including AlAs binary layers in the bottom DBR). For the two designs, within our experimental error, most key triple array results (*L*_max_, maximum *f*_3dB_, D factor, MCEF, maximum error free bit rate, etc.) were nearly the same for equal *ϕ*, indicating that the binary DBR layers (as expected) enable increased *L*_max_ at rollover compared to purely ternary DBR layers. As we increase the number of top-emitting VCSEL elements, the heat dissipating advantages of the binary Kapalua design will likely more clearly emerge, although we believe clever packaging schemes are equally if not more important.

We anticipate that our work will help lead to compact, low-cost, temperature-stable, and reliable lasers for 5G and 6G systems applications with flexible geometry and scalable optical output power (e.g., by increasing the number of lasing elements in an array). We work at 980 nm for convenience, whereas our results and conclusions apply equally well to VCSEL arrays emitting from the ultraviolet to well above 1550 nm. Wavelength selection for practical systems depends typically on the desired photodetector (e.g., 905–910 nm for shallow silicon charge coupled devices, 940 nm for silicon avalanche photodiodes (PDs), and 980 nm for InGaAs PDs), and eye safety, which may also depend on the required free-space beam shape, for example, a pencil beam versus a conical beam.

## Figures and Tables

**Table 1:** Extracted |S21| fitting parameters for the Kapalua triple 980 nm VCSEL with *ϕ*~6 μm.

DC Bias (mA)	*J* (kA/cm^2^)	*~ϕ* (µm) Each VCSEL	*f_R_* (GHz)	*f_p_* (GHz)	*γ* (ns)	*f*_3dB_ (GHz)	DC *L* (mW)
4.0	4.7	6.0	10.56701	7.52671	26.87444	13.0	2.4
8.5	10.0	6.0	15.63646	10.52691	38.10489	19.1	6.5
18.5	21.8	6.0	21.66922	13.91861	67.64717	25.2	15.1

## Data Availability

The data presented in this study are available on request from the corresponding author.
